# Mapping of neuronal redox conditions in a mouse model of Rett syndrome

**DOI:** 10.1016/j.ynirp.2025.100297

**Published:** 2025-10-24

**Authors:** Hendrik Woeste, Laura van Agen, Michael Müller

**Affiliations:** Universitätsmedizin Göttingen, Institut für Neuro- und Sinnesphysiologie, Humboldtallee 23, D-37073, Göttingen, Germany

**Keywords:** Oxidative stress, Reactive oxygen species, Redox imaging, roGFP, Redox imbalance, Mouse brain, MeCP2 deficiency, Mitochondrial catalase

## Abstract

Rett syndrome (RTT) is associated with a systemic redox imbalance, potentially provoking cellular dysfunction and contributing to some of the disease symptoms. While previous studies have reported these redox alterations also in brain, the exact cerebral redox pattern remains unclear. We therefore generated MeCP2-deficient mice expressing the cytosolic redox sensor roGFPc in excitatory projection neurons. Taking advantage of the earlier developed excitation ratiometric 2-photon imaging, we mapped the redox conditions of individual hippocampal and cortical neurons in acute brain tissue slices of female mice. These quantitative redox analyses revealed clear brain-regional differences in the degree of roGFPc oxidation, with dentate gyrus and CA3 being most oxidized, CA1 being least oxidized and cortical areas presenting intermediate oxidation levels. On postnatal day p50, hardly any RTT-related differences were evident. With maturation (>p100), redox conditions became more reducing in WT females. This was, however, not the case in MeCP2-deficient females, whose hippocampal and especially cortical neurons now appeared clearly more oxidized. By correlative redox microscopy, we succeeded to relate cellular redox-conditions to cellular MeCP2 expression. Validation in CA1 and somatosensory cortex revealed that, based on improved discrimination sensitivity, a more oxidized redox balance became detectable in MeCP2-deficient cortical neurons already on p50. Expression of a mitochondrial catalase efficiently abolished the more oxidizing redox milieu in MeCP2-deficient cortical neurons. This confirms a widespread oxidative burden in forebrain neurons, which manifests already in pre-symptomatic MeCP2-deficient female mice and intensifies with disease progression. Stabilizing mitochondrial function by targeted catalase expression proved potentially protective.

## Highlights

• Redox imaging revealed more oxidized neuronal redox conditions in various brain regions of female *Mecp2*^*+/−*^ mice beyond p100.
• Whereas a maturational reducing shift occured in WT mice, this was not the case in *Mecp2*^*+/−*^ females.
• Correlation with MeCP2 expression proved already on p50 more oxidized redox conditions in MeCP2-deficient cortical neurons.
• Expressing a mitochondrial catalase abolished these cellular redox differences, suggesting a potential merit of this treatment.

## List of abbreviations

3V: third ventricle
ACSF: artificial cerebrospinal fluid
Au1: primary auditory cortex
AuD: secondary auditory cortex, dorsal area
AuV: secondary auditory cortex, ventral area
CA1: *cornu ammonis* subfield 1
CA3: *cornu ammonis* subfield 3
D3V: dorsal third ventricle
DG: dentate gyrus
DTT: 1,4-dithio-DL-threitol
Ect: ectorhinal cortex
F_740_/F_910_: roGFP fluorescence ratio
F910_ox_/F910_red_: ratio of fluorescence intensities (instrument factor)
LPtA: lateral parietal association cortex
LV: lateral ventricle
M1: primary motor cortex
M2: secondary motor cortex
mCAT: mitochondrial catalase
*MECP2*: human gene encoding methyl-CpG-binding protein 2
*Mecp2*: mouse gene encoding methyl-CpG-binding protein 2
MeCP2: methyl-CpG-binding protein
MPtA: medial parietal association cortex
OxD_roGFPc_: relative degree of roGFPc oxidation
p50: postnatal day 50
PBS: phosphate buffered saline
PFA: paraformaldehyde
PMT: photomultiplier tube
R: fluorescence ratio (F_740_/F_910_)
roGFP1: reduction-oxidation sensitive green fluorescent protein 1
roGFPc: cytosolically-expressed roGFP1
ROS: reactive oxygen species
R_ox_: fully oxidized fluorescence ratio
R_red_: fully reduced fluorescence ratio
RSA: retrosplenial agranular cortex
RSG: retrosplenial granular cortex
RT: room temperature
RTT: Rett syndrome
S: subiculum
S1: primary somatosensory cortex
S1BF: primary somatosensory cortex, barrel field
S1HL: primary somatosensory cortex, hindlimb region
S1Tr: primary somatosensory cortex, trunk region
S2: secondary somatosensory cortex
TeA: temporal association cortex
Thy-1.2: thymocyte differentiation antigen 1
Ti:Sa: titanium-sapphire
TPLSM: 2-photon laser scanning microscope
V1: primary visual cortex
V2L: secondary visual cortex, lateral area
V2ML: secondary visual cortex, mediolateral area
V2MM: secondary visual cortex, mediomedial area
WT: wildtype

## 1 Introduction

Rett syndrome (RTT) is an X-chromosome linked neurodevelopmental disorder, which is manifesting primarily in females during early childhood (Hagberg et al., 1983; Rett, 1966). RTT typically arises from *de novo* mutations in the *MECP2* gene which is encoding the transcriptional regulator methyl-CpG-binding protein 2 (MeCP2) (Amir et al., 1999). As a result of these mutations, the function of MeCP2 is impaired, with downstream consequences for several other genes and the coded proteins (Zlatic et al., 2023). As the expression level of MeCP2 is particularly high in neurons of the maturing brain (Shahbazian et al., 2002), the limited MeCP2 function culminates in an array of in particular neurological/neuropsychiatric symptoms accompanied by physical disabilities. These typically include, cognitive impairment, loss of speech, seizures, motor dysfunction with hand stereotypies, weight loss, scoliosis, and cardio-respiratory irregularities [see (Chahrour and Zoghbi, 2007)].

One aspect of this complex clinical manifestations is systemic oxidative stress, which is evident as increased lipid-peroxidation, lowered superoxide-dismutase levels, intensified protein carbonylation and increased non-protein-bound iron levels in patient blood samples (De Felice et al., 2009; Sierra et al., 2001). Furthermore, signs of intensified membrane/myelin damage have been detected (De Felice et al., 2011), suggesting that the oxidative burden affects also the brain of patients. Indeed, in *Mecp2*-mutant mice such cerebral redox-alterations could be confirmed unequivocally (De Felice et al., 2014; De Filippis et al., 2015; Groβer et al., 2012). However, a more detailed understanding of which brain regions or even which specific cell types are particularly affected by these redox alterations is still sparse.

By taking advantage of our transgenic redox-indicator mice (Wagener et al., 2016), which stably express the genetically encoded redox-indicator roGFP1 (reduction oxidation sensitive green fluorescent protein 1) (Hanson et al., 2004), we mapped baseline redox conditions in the hippocampus and cortex of MeCP2-deficient mice. With roGFP being expressed in the cytosol of excitatory projection neurons, we thereby extended redox analyzes to the cellular and even subcellular level, to decipher redox alterations in specific brain regions and defined neurons. Our analyses were conducted on acute brain tissue slices from female mice to define the redox balance under the conditions of X-chromosomal mosaicism, which reflects the clinical conditions in patients with RTT. To take disease progression into account, female mice were studied around postnatal day p50, when *Mecp2*^*+/*^**^*−*^** mice are still largely presymptomatic, and beyond p100, when *Mecp2*^*+/*^**^*−*^** mice develop first symptoms (Guy et al., 2001). In addition to the cerebral redox mapping, we developed a correlative microscopical approach to refer neuronal redox conditions to the extent of cellular MeCP2 expression. In a first assessment, it proved to be extremely useful, especially under the conditions of X-chromosomal mosaicism in heterozygous *Mecp2*^*+/*^**^*−*^** mice. This also included analyses of MeCP2-deficient mice in which a mitochondrial catalase (mCAT) was expressed as an attempt to stabilize cellular redox-buffering.

## 2 Materials and methods

### 2.1 Preparation

As a mouse model for Rett syndrome, we chose the severely affected *Mecp2* knockout model generated by Adrian Bird [B6J.129P2(C)-*Mecp2*^*tm1.1Bird*^] (Guy et al., 2001). Heterozygous breeding females (*Mecp2*^*+/−*^) were purchased from the Jackson Laboratories and mated with male C57BL6/J mice at the central animal facility of the University Medical Center Göttingen. To obtain *Mecp2*-mutant mice that also express the cytosolic redox indicator roGFP, female *Mecp2*^*+/−*^ mice were bred with male *roGFPc*^*+/T*^ mice of our redox-indicator mouse strain (B6J-Tg(Thy1.2-roGFP1c)2Mmllr) (Wagener et al., 2016), giving rise to the double transgenic *Mecp2_roGFPc* mice [B6J.129P2(C)-*Mecp2*^*tm1.1Bird*^ Tg(Thy1.2-roGFP1c)2Mmllr] (Müller, 2019). On a few instances and meant as treatment approach, these double transgenic mice were crossbred with the mCAT mouse line [Tg(CAG-OTC/CAT)4033Prab] expressing a mitochondrial catalase (Schriner et al., 2005), which were obtained from the Jackson Laboratories. Thereby, we obtained triple transgenic *Mecp2_mCAT_roGFPc* mice [B6.129P2(C)-*Mecp2*^*tm1.1Bird*^ Tg(CAG-OTC/CAT)4033Prab Tg(Thy1.2-roGFP1c)2Mmllr]. Breeding of mouse strains and all related animal procedures were in accordance to national German and European regulations and were approved by either the Lower Saxony State Office for Consumer Protection and Food Safety (files G16/2177, G17/2544, G21/3658) or the Office of Animal Welfare of the University Medical Center Göttingen (files T13/08, T22/34).

Acute cortico-hippocampal tissue slices were obtained from female *Mecp2_roGFPc* or *Mecp2_mCAT_roGFPc* mice. In detail, deeply anesthetized mice (diethylether or isoflurane) were decapitated, the brain was rapidly but gently isolated and immediately cooled down in ice-cold artificial cerebrospinal fluid (ACSF, composition see below) for ∼3 min. In total, 4 coronal tissue slices of 400 μm thickness were prepared (752M Vibroslice, Campden Instruments) from each mouse, choosing slices from the more rostral and the more caudal positions of the hippocampal formation (see Fig. 2). These slices were then separated along their respective sagittal midlines, and the hemi-slices rested for a minimum of 90 min in a submersion-style storage chamber with oxygenated ACSF (20–22 °C) before the redox imaging experiments.

Two-photon imaging was conducted in a submersion style chamber, which was custom-made by our mechanical workshop to meet the specific technical requirements of our experiments. The chamber was heated to ∼30.0 °C and constantly supplied with fresh oxygenated ACSF at a rate of ∼4 ml/min.

### 2.2 Solutions

All drug compounds and chemicals were obtained from Sigma-Aldrich, unless stated differently. ACSF contained (in mM): 130 NaCl, 24 NaHCO_3_, 3.5 KCl, 1.25 NaH_2_PO_4_, 1.2 CaCl_2_, 1.2 MgSO_4_, and 10 dextrose. All solutions were constantly aerated with carbogen (95 % O_2_, 5 % CO_2_) to maintain pH 7.4. DTT (1,4-dithio-DL-threitol) and H_2_O_2_ (hydrogen peroxide) were directly added to ACSF. H_2_O_2_ was obtained as aqueous 30 % solution of which fresh 1 M working stocks were prepared daily.

### 2.3 Optical recordings

**Fig. 1:**
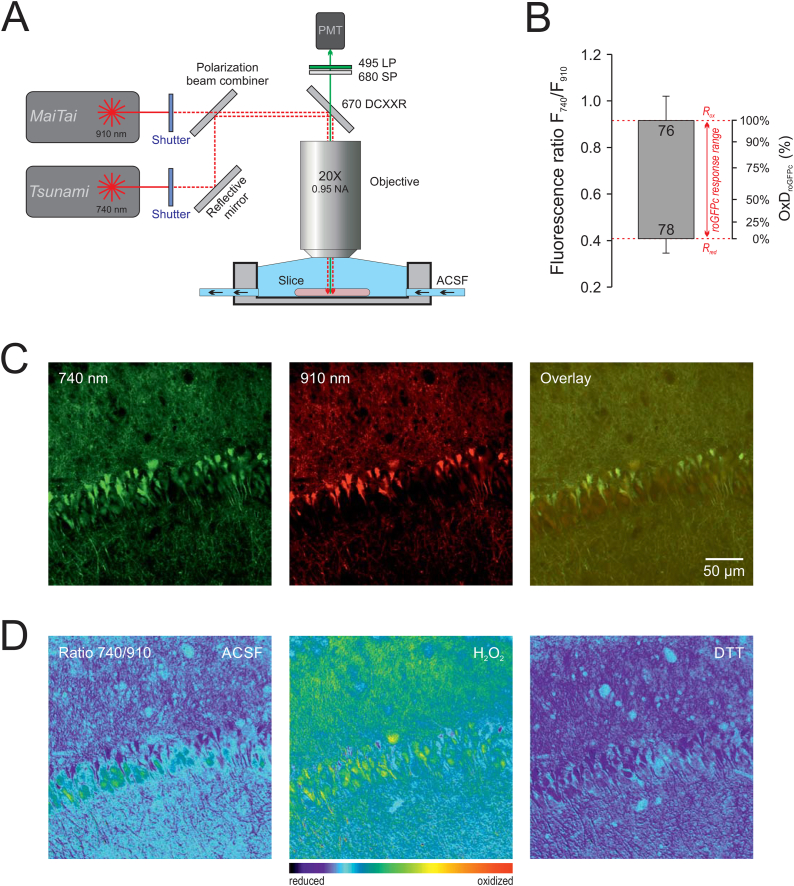
Excitation ratiometric dual-laser based 2-photon redox imaging. **(A)** Schematic imaging system layout. Each laser is tuned to one of the desired excitation wavelengths and the discrete laser beams are aligned by a polarization beam combiner. Alternate excitation is realized by mechanical shutters. **(B)** Calibrated roGFPc response range as determined in CA1 pyramidal neurons. The upper end of the box corresponds to the fully oxidized ratio induced by H_2_O_2_ treatment (10 mM, 7 min), the lower end indicates the fully reduced state induced by DTT (5 mM, 7 min). Error bars represent standard deviations and the number of cells analyzed is indicated. **(C)** Ratiometric image pair consisting of the individual images of CA1 pyramidal neurons excited at the discrete wavelengths of 740 nm and 910 nm as well as the resulting overlay image. **(D)** Ratiometric images computed offline from the ratiometric image pair of panel C. Displayed are the resting baseline control conditions in ACSF as well as the fully oxidized and the fully reduced state elicited by H_2_O_2_ and 5 mM DTT, respectively. Ratiometric values are represented in a 12 bit pseudocolor scale (4096 levels) with warm colors representing oxidation and cold colors representing reduction.

To combine the excitation-ratiometric properties of roGFP with 2-photon imaging, we took advantage of our earlier established approach of dual-laser based excitation ratiometric 2-photon imaging (Wagener et al., 2016). In detail, our TriMScope II 2-photon laser scanning system (LaVision BioTec) was equipped with two Ti:Sa laser systems (Millennia eV10 Prime-pumped Tsunami and MaiTai eHP DS, Newport-Spectra Physics). The two lasers are coupled - each via a separate, shutter-controlled optical port - into the scanhead and then aligned by a polarization beam-combiner. For details and filter specifications see Fig. 1A.

**Fig. 2:**
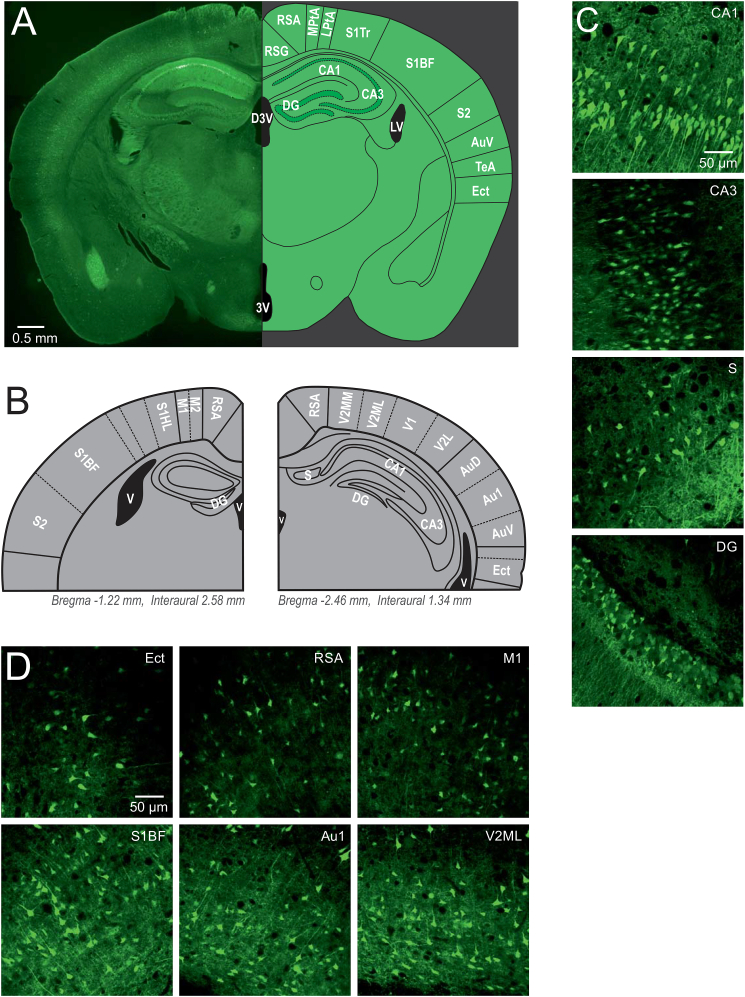
Cerebral expression of roGFPc in excitatory projection neurons. **(A)** Based on the expression pattern, redox mapping was performed in CA1, CA3, DG and subiculum (S) of the hippocampus as well as in visual, auditory, ectorhinal (Ect), somatosensory (S1), motor (M1), and retrosplenial agranular cortex (RSA). **(B)** Schematic of the analyzed slices chosen from a more rostral and a more caudal position to include the desired brain regions. Approximate rostrocaudal positions are indicated for each drawing. **(C)** Sample images of the hippocampal subfields. **(D)** Sample images of different cortical regions studied. Scaling is identical for all subfields displayed.

In acute tissue slices, the intended brain regions were located with a 5X 0.13NA low magnification objective (Epiplan, Zeiss) and 490 nm epifluorescence excitation (pE1, CoolLED). For quantitative redox imaging of individual neurons, a 20X 0.95NA (XLUMPlanFI, Olympus) objective was used. Ratiometric image pairs were taken by alternate 740 nm and 910 nm 2-photon excitation, based on which overlay and ratiometric images (F_740_/F_910_) were then calculated (Metamorph Offline 7.5, Molecular Devices). The redox-ratio was determined (averaged) within circular regions of interest (ROIs) placed in the soma region of clearly identifiable, roGFP-expressing neurons. For visualization purposes, an additional scaling factor of 1000 was introduced [R = (F_740_ ∗ 1000)/F_910_] to make reasonable use of the ratiometric 12-bit pseudocolor palette. An increased fluorescence ratio F_740_/F_910_ indicates oxidation of roGFP, a decreased ratio indicates reduction of roGFP.

True quantitative redox analyses require calibration of the ratiometric roGFPc response range under the specific experimental conditions. In the CA1 pyramidal cell layer we therefore determined the fluorescence ratios representing full oxidation (R_ox_) and full reduction as well as the ratio of fluorescence intensities (F910_ox_/F910_red_). The latter is also referred to as instrument factor and is based on the wavelengths, intensity setting and optical parameters of the respective imaging system. For full oxidation, the given slice was exposed to 5 mM H_2_O_2_ (7 min) and upon proper wash-out for ∼25 min the very same slice was then exposed to 10 mM DTT (7 min) to induce full reduction. Based on the calibration parameters, the relative degrees of roGFP oxidation (OxD_roGFPc_) were then calculated from the fluorescence ratios R determined in the different types of roGFPc-expressing neurons under our recording conditions (Meyer and Dick, 2010; Wagener et al., 2016):

(equation 1) $${\text{OxD}}_{\text{roGFPc}}=\frac{R-R_{\text{red}}}{\frac{{F910}_{\text{ox}}}{{F910}_{\text{red}}}\;\left(R_{ox}-R\right)+\left(R-R_{red}\right)}$$

### 2.4 Correlative redox microscopy

Immediately after redox imaging, laser marks (∼50 × 50 μm) were bleached into the slice next to the imaged tissue regions to facilitate their later re-identification (see Fig. 4B), before the brain tissue slices were fixed (4 % PFA in phosphate buffered saline, PBS, 3 h, room temperature, RT). Afterwards, the slices were washed three times in PBS (10 min each) and then kept refrigerated in PBS (4 °C). Right before staining, the slices were quenched in NH_4_Cl (100 mM, 30 min, RT) and blocked in BSA (1 %, 1 h). Next, they were incubated for 3–4 days in 1 % BSA and 0.4 % Triton-X-100 with the primary anti-MeCP2 antibody (MeCP2 (D4F3) XP rabbit mAB, Cell Signaling Technology, #3456; 1:1000 dilution, 4 °C). Slices were then washed 3 times at RT (0.1 % Triton-X100 in PBS, 10 min each) and incubated with the secondary antibody (donkey anti-rabbit Dylight 594; lot:XE350792, 1:2000 dilution, 3 h, RT). Upon another round of 3 × 10 min washes with PBS, nuclear labeling was performed with Hoechst 33342 (ThermoFisher Scientific; #62249; 1:10.000 dilution; 30 min, RT). After final washing (3 × 10 min, PBS, RT), the labeled slices were kept in PBS at 4 °C in the dark until imaging was performed. All washing and incubation steps were performed under gentle shaking.

Hoechst 33342 and roGFPc fluorescence were excited at 740 nm and 910 nm respectively, and the resulting emissions separated by an emission dichroic (DC 495) with additional blue (465/30 nm, Hoechst) and green bandpass filters (525/50 nm, roGFP). MeCP2-Dylight 594 fluorescence was excited at 1040 nm and separated from the GFP emission by an emission dichroic (DC 570 nm) and a red bandpass filter (617/73 nm). For triple-label imaging, Z-stacks of 10–20 optical planes (2 μm steps) were recorded for each condition. During post-experimental analysis, the best optical plane was chosen from these stacks and the respective overlay images computed.

### 2.5 Statistics

Reported data were collected from 22 female mice, typically using up to 4 slices from each brain. To ensure the independence of observations, each experimental paradigm was conducted on at least 3 different mice. For a first screening of data, an outlier test (ROUT, Q = 1 %, GraphPad Prism 9.5.1.) was performed. Results are reported as mean ± standard deviation, unless stated differently, and n represents the number of neurons analyzed. Statistical differences in the redox conditions among the different age levels and genotypes were assessed by two way ANOVA (analysis of variance) followed all by pairwise Holm-Šídák comparisons. In the diagrams, significant differences between young and older mice are marked by asterisks (∗p < 0.05, ∗∗p < 0.01) whereas genotype-related differences among WT and *Mecp2*^*+/−*^ mice are indicated by cross-hatches (#p < 0.05, ##p < 0.01); the underlying statistical tests are specified. For data processing and statistical testing we used Excel (Office, 2016), GraphPad Prism 9.5.1 (GraphPad, Inc.) and Sigma Stat 3.5 (Systat Software).

## 3 Results

In this study we aimed to map the redox state in different brain regions of female *Mecp2*-mutant (*Mecp2*^*+/−*^) mice. This became possible by cross-breeding our earlier generated redox indicator mice (Wagener et al., 2016) with *Mecp2*-mutant mice (Guy et al., 2001), thereby obtaining double transgenic *Mecp2_roGFPc* mice [B6J.129P2(C)-*Mecp2tm*^*1.1Bird*^ Tg(Thy1.2-roGFP1c)2Mmllr] (Müller, 2019).

To take the disease progression into account, the quantitative redox mapping was performed at two different age levels of p50 (range p50-p60) and beyond p100 (range p108-p307), directly comparing WT and *Mecp2*^*+/−*^ siblings. Ratiometric image pairs were taken ∼50 μm below the slice surface, to exclude those superficially located neurons being damaged during the slicing process. To guarantee stable recording conditions, it turned out crucial to optimize laser fine-adjustment and alignment of the two laser beams on a daily basis. This also included the use of fluorescent 10 μm beads (Fluoresbrite YG 10.0 μm, Polysciences) as reference specimen to ensure that the ratiometric readings were stable and within the desired range.

### 3.1 Redox sensor calibration

The quantitative redox analyses are made possible by the excitation-ratiometric properties of roGFP (Hanson et al., 2004). However, they require response range calibrations of the respective redox sensor applied for the given experimental conditions (Funke et al., 2011; Meyer and Dick, 2010). These calibrations of roGFPc were performed in *stratum pyramidale* of the hippocampal CA1 subfield, as it showed the highest density of roGFPc expressing neurons. Exposing the slices to H_2_O_2_ (5 mM, 7 min treatment) indicated a fully oxidized ratio R_ox_ of 0.916 ± 0.106 (n = 76 cells), and upon proper wash-out the exposure to DTT (10 mM, 7 min treatment) provided and a fully reduced ratio R_red_ of 0.408 ± 0.063 (n = 78 cells). In addition, an instrument factor F910_ox_/F910_red_ of 0.497 ± 0.126 was obtained. Based on these calibration parameters, the roGFPc fluorescence ratios F_740_/F_910_ determined for the individual neurons, were converted (see equation (1)) into the relative degrees of GFPc oxidation (OxDroGFPc), which represent a true quantitative measure of redox conditions in the different brain regions, age levels and genotypes.

### 3.2 Neuronal redox mapping

The redox mapping performed was based on the expression pattern of roGFP in excitatory projection neurons of our redox-indicator mice (Fig. 2), with a focus on hippocampus and cortex. In detail, we assessed CA1, CA3, dentate gyrus (DG), and subiculum (S) of the hippocampus as well as visual cortex (VIS), auditory cortex (AUD), somatosensory cortex, motor cortex (M), retrosplenial agranular cortex (RSA) and ectorhinal cortex (Ect). The visual cortical regions analyzed included neurons from primary visual cortex (V1), as well as the lateral (V2L), mediolateral (V2ML) and mediomedial areas of secondary visual cortex (V2MM). The auditory cortex included neurons from primary auditory cortex (Au1), as well as from the dorsal (AuD) and ventral (AuV) areas of secondary auditory cortex. The mapping of somatosensory cortex included primary somatosensory cortex (S1), its barrel field (S1BF), the hindlimb (S1HL) and trunk region (S1Tr), as well as secondary somatosensory cortex (S2). For motor cortex we included neurons from primary (M1) and secondary (M2) motor cortex; DG represents its upper and lower blade (Fig. 2).

The resulting ratiometric images contained numerous cells, each of which was assessed for intactness and provided a ratiometric value of the redox baseline. As explained in Material and Methods, the calculated roGFPc redox ratios were converted into the relative degrees of roGFP oxidation (OxDroGFPc), to allow for a reasonable comparison of brain regions, age levels and genotypes. As reasonable range and intact neurons we considered an OxDroGFPc in between 5 and 95 %. In total, we quantified the resting redox baseline conditions of 4569 neurons (8 outliers identified and removed), thereby comparing WT and *Mecp2*^*+/−*^ mice at age ∼ p50 and >p100.

**Fig. 3:**
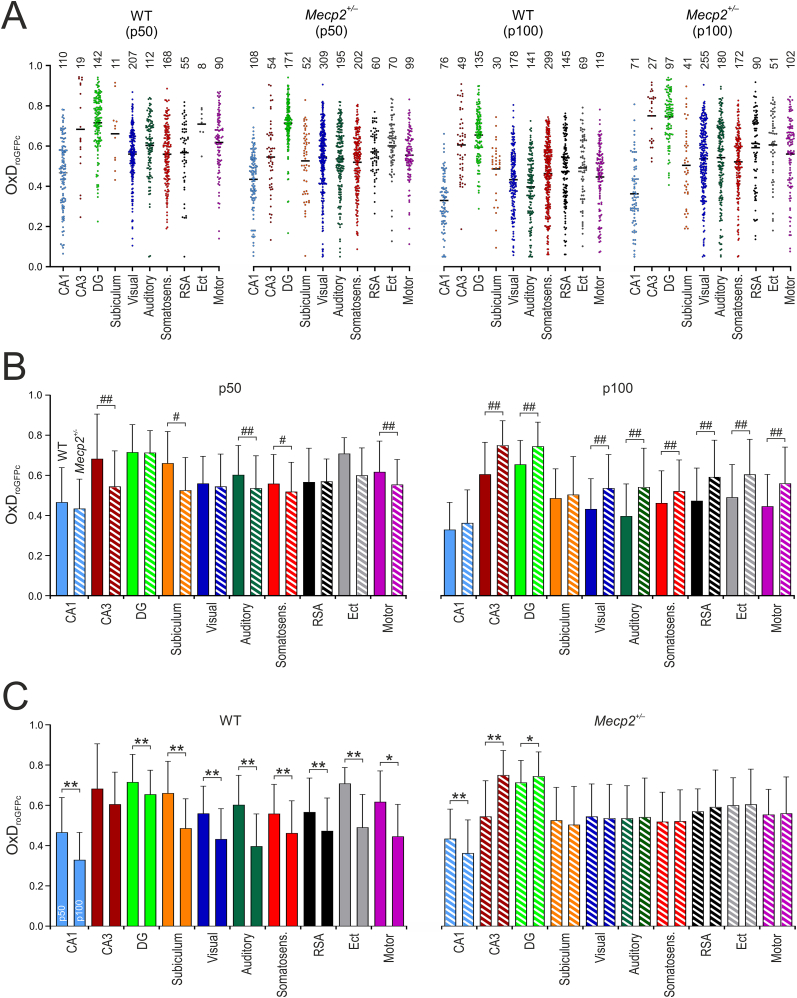
Neuronal redox balance in the hippocampal and cortical subfields. **(A)** Dot plots summarizing the redox conditions in young adult (p50) and more mature (>p100) female mice. Marked differences in redox balance are obvious among the different brain regions. Plotted is the relative degree of roGFPc oxidation (OxDroGFPc), with each dot representing an individual neuron. Black line indicates the mean, and the number of neurons is indicated above each group. **(B)** Genotypic comparison of redox conditions in WT and *Mecp2*^*+/*^**^*−*^** mouse brains. Plotted values represent mean ± standard deviation. Widespread genotype-related differences with more oxidized conditions in *Mecp2*^*+/*^**^*−*^** mice become evident in more mature animals (>p100). Solid bars represent WT mice, hatched bars represent *Mecp2*^*+/*^**^*−*^** mice. **(C)** Age-related changes in neuronal redox conditions. Whereas redox-balance in WT mice becomes more reducing with maturation, this redox-shift does not occur in *Mecp2*^*+/*^**^*−*^** mice. The left bar of each pair represents p50, the right bar of each pair represents p100. Statistical differences among ages and genotypes were assessed by two-way ANOVA followed by all-pairwise Holm-Šídák comparisons. Significant differences among p50 and p100 are indicated by asterisks (∗p < 0.05, ∗∗p < 0.01) and those among WT and *Mecp2*^*+/*^**^*−*^** mice are identified by crosshatches (#p < 0.05, ##p < 0.01).

Clear brain-regional differences were obvious in the resting redox conditions within the different brain regions. In general, CA1 showed the lowest OxDroGFPc, whereas CA3 as well as DG exhibited most oxidized conditions (Fig. 3A and B). For example, in p50 WT females CA1 pyramidal neurons showed an average OxDroGFPc of 0.466 ± 0.172 (n = 110), whereas in granule neurons of the dentate gyrus it averaged 0.719 ± 0.132 (n = 142). These regional differences were evident in WT and *Mecp2*^*+/−*^ mice at both maturational stages (Fig. 3A and B).

**Fig. 4:**
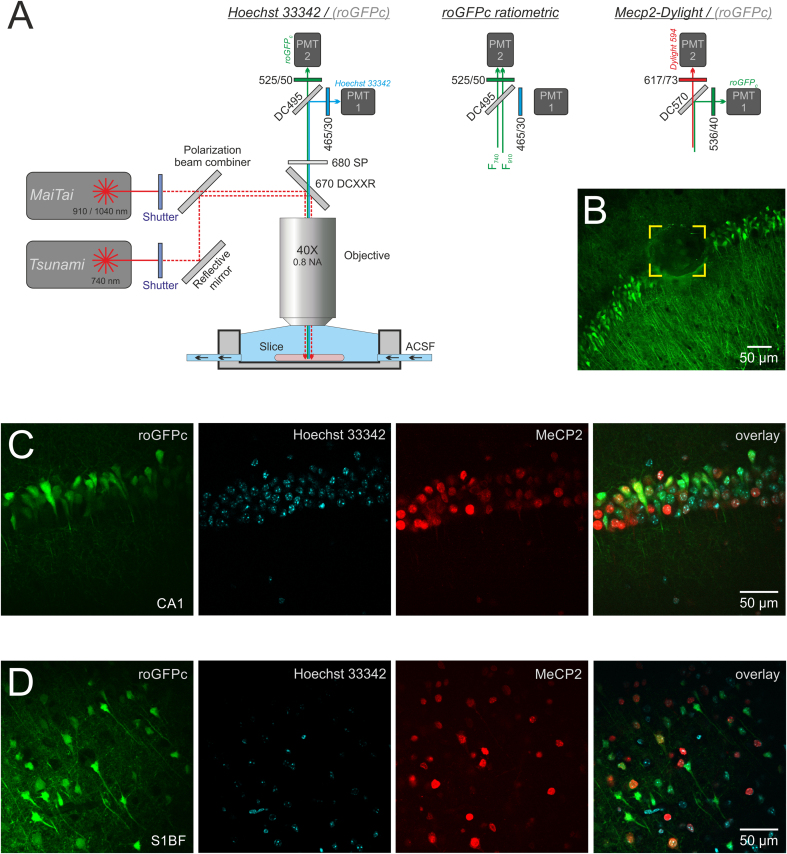
Relating cellular redox-balance to MeCP2 expression by correlative redox microscopy. **(A)** Technical settings of the different modes of 2-photon imaging applied, i.e. imaging of nuclear labels (Hoechst 33342), MeCP2 expression (Dylight 594) and ratiometric redox imaging (roGFPc). As indicated, besides adjusting the MaiTai wavelengths (either 910 or 1040 nm) further filter modifications were required in the emission light path to ensure in the triple-labeled slices an efficient separation of Hoechst 33342, Dylight 594 and roGFPc fluorescence into the desired detection channels. **(B)** Corticohippocampal slice viewed at lower resolution to show the laser bleach mark – flanked by the yellow brackets – within the pyramidal cell layer. Such marks served to re-identify the earlier imaged slice regions. **(C)** Image-set obtained by correlative redox microscopy from the hippocampal CA1 subfield of a female *Mecp2*^*+/*^**^*−*^** mouse (p50). As evident from the overlay, these images allow to refer neuronal redox balance (roGFP) to the extent of MeCP2 expression in the respective nuclei (Hoechst 33342). **(D)** Corresponding image set recorded from *Mecp2*^*+/*^**^*−*^** somatosensory cortex (p50). Note the irregular distribution of MeCP2-positive cells.

The RTT related genotypic differences in OxDroGFPc were less pronounced in the young, still presymptomatic mice. At p50, CA3 and subiculum of *Mecp2*^*+/−*^ mice were even less oxidized than in WT, whereas CA1 and DG did not differ among genotypes. In addition, auditory, somatosensory, and motor cortex were less oxidized in *Mecp2*^*+/−*^ than in WT females, whereas visual cortex, RSA, and Ect did not differ among genotypes.

This clearly changed with maturation (Fig. 3C). Beyond postnatal day p100, CA3 and DG were more oxidized in *Mecp2*^*+/−*^ than in WT females; CA1 and subiculum did not differ among genotypes. In all cortical regions analyzed, i.e. visual, auditory, somatosensory, RSA, Ect and motor cortex, significantly more oxidized conditions were present in *Mecp2*^*+/−*^ mice. Closely inspecting the different age groups revealed that in WT females neuronal redox conditions became more reducing with maturation (Fig. 3C). Interestingly, this reducing shift – with the exception of CA1 – did not occur in *Mecp2*^*+/−*^ mice, whose neurons in consequence became more oxidized than those in WT mice.

In addition to performing statistical redox analyses on the cellular level, we averaged the redox conditions for all neurons within a studied brain slice to enable a comparison at the tissue slice level. The respective results are summarized in Table 1. They are somewhat less pronounced, but also indicate the general reducing shift with maturation of WT mice as well as more oxidized redox conditions in DG, visual, auditory, RSA, Ext and motor cortex of *Mecp2*^*+/−*^ mice beyond p100.

**Table 1:** Summary of neuronal redox-baselines in different brain regions and genotypes of young adult (p50) and more mature (>p100) mice.

Brain Region	WT (p50)	*Mecp2*^*+/−*^ (p50)	WT (>p100)	*Mecp2*^*+/−*^ (>p100)
CA1	0.452 ± 0.121 (n = 10)	0.428 ± 0.056 (n = 9)	0.337 ± 0.038 (n = 9) **∗**	0.409 ± 0.148 (n = 6)
CA3	0.719 ± 0.206 (n = 7)	0.664 ± 0.192 (n = 7)	0.689 ± 0.145 (n = 12)	0.770 ± 0.085 (n = 5)
DG	0.747 ± 0.082 (n = 10)	0.721 ± 0.037 (n = 10)	0.660 ± 0.054 (n = 9) **∗∗**	0.743 ± 0.070 (n = 6) **#**
Subiculum	0.699 ± 0.206 (n = 3)	0.551 ± 0.128 (n = 8)	0.540 ± 0.124 (n = 6)	0.557 ± 0.154 (n = 6)
Visual CTX	0.568 ± 0.041 (n = 7)	0.557 ± 0.081 (n = 8)	0.446 ± 0.074 (n = 5) **∗∗**	0.549 ± 0.082 (n = 6) **#**
Auditory CTX	0.632 ± 0.054 (n = 7)	0.535 ± 0.071 (n = 8) **#**	0.411 ± 0.103 (n = 8) **∗∗**	0.537 ± 0.062 (n = 6) **##**
Somatosens. CTX	0.593 ± 0.097 (n = 8)	0.525 ± 0.051 (n = 6)	0.465 ± 0.087 (n = 11) **∗∗**	0.518 ± 0.075 (n = 6)
RSA CTX	0.571 ± 0.105 (n = 7)	0.573 ± 0.042 (n = 6)	0.493 ± 0.108 (n = 13)	0.621 ± 0.134 (n = 12) **##**
Ect CTX	0.702 ± 0.028 (n = 3)	0.621 ± 0.062 (n = 9)	0.495 ± 0.091 (n = 9) **∗∗**	0.610 ± 0.109 (n = 7) **#**
Motor CTX	0.628 ± 0.111 (n = 5)	0.553 ± 0.028 (n = 5)	0.450 ± 0.046 (n = 5) **∗∗**	0.575 ± 0.134 (n = 6) **#**

Neuronal redox baselines are expressed as relative degrees of roGFPc oxidation (OxD_roGFPc_) and were averaged on the tissue slice level. Plotted data represent means ± standard deviations; the number of slices (n) analyzed is reported. Results were obtained from 3 to 4 female mice per group. In some brain regions (S, Ect), sufficient expression levels were not found in each mouse studied. Statistical differences among ages and genotypes were assessed by two-way ANOVA followed by all-pairwise Holm-Šídák comparisons. Significant differences among age-groups are indicated by asterisks (∗p < 0.05, ∗∗p < 0.01) and those among WT and *Mecp2*^*+/*^**^*−*^** mice are indicated by crosshatches (#p < 0.05, ##p < 0.01).

### 3.3 Correlating redox conditions with MeCP2 expression

In view of the more oxidized redox-conditions that manifested among older *Mecp2*^*+/−*^ and WT mice, we were wondering whether the X-chromosomal mosaicism present in female *Mecp2*^*+/−*^ mice might give rise to detectable redox differences among MeCP2 expressing and MeCP2 deficient neurons. This would, however, require to correlate cellular redox conditions with the expression of MeCP2 on the single cell level in vital tissue. To solve this challenge, we developed a correlative redox microscopy approach (Fig. 4). Acute brain tissue slices first underwent ratiometric redox imaging, and were then immediately fixed (4 % PFA) for subsequent anti-MeCP2-labeling (Cell Signaling Technology, #3456) and nuclear staining (Hoechst 33342). Upon successful staining, the previously redox-imaged neurons were re-identified based on laser “bleaching-marks” intentionally placed next to the redox-imaged regions (Fig. 4B), and were then imaged once more to determine their extents of MeCP2 expression.

We assessed the suitability of this approach in the hippocampal CA1 subfield and in somatosensory cortex, as these regions showed a particular high density of roGFP-expressing neurons. Chosen were female *Mecp2*^*+/−*^ mice at p50, which in the initial set of redox-imaging analyses did not present any oxidative redox shifts yet.

Since a 40x 0.8 NA objective (IR-Achroplan Zeiss) was used and slightly different emission filter configurations were required in this second set of imaging experiments (Fig. 4A), the earlier conducted roGFPc calibrations could not be applied to these particular optical conditions. Instead, for a reliable comparison, we normalized the roGFPc fluorescence ratios determined in MeCP2-deficient neurons to the averaged redox conditions measured in MeCP2-positive neurons of the given brain tissue slice.

**Fig. 5:**
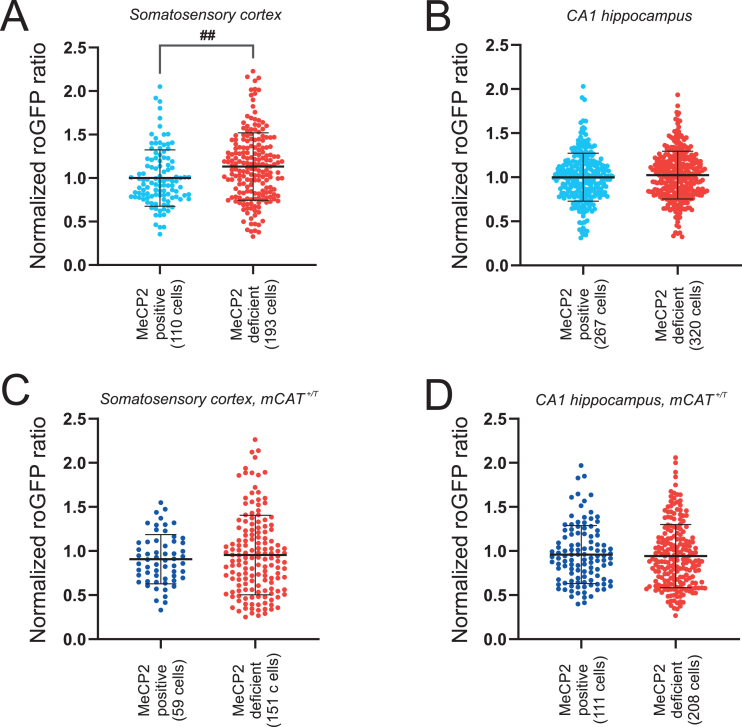
Mosaicism of MeCP2 expression in female *Mecp2*^*+/*^**^*−*^** mice gives rise to redox mosaicism. **(A)** In somatosensory cortex of presymptomatic *Mecp2*^*+/*^**^*−*^** mice (p50), MeCP2-deficient neurons showed more oxidized redox-baselines than MeCP2-positive neurons (Welsh's *t*-Test, ##p < 0.01). **(B)** In the hippocampal CA1 subfield such redox-differences were not evident. **(C)** Expression of a mitochondrial catalase (mCAT) – delivered by crossbreeding – abolished these redox-differences in somatosensory cortex. **(D)** With mCAT expressed, MeCP2-deficient and MeCP2-positive CA1 hippocampal neurons still did not differ in their redox-conditions. Plotted are normalized roGFPc ratios, obtained by referring (dividing) the roGFP fluorescence ratio (F_740_/F_910_) of MeCP2-deficient neurons to the averaged roGFPc fluorescence ratio of MeCP2-positive neurons in the respective brain tissue slice.

In somatosensory cortex a total of 303 neurons was re-identified successfully by this approach of correlative microscopy, 36 % of which expressed MeCP2. Indeed, the cellular comparison revealed slightly more oxidized redox conditions in MeCP2-deficient cortical neurons (1.101 ± 0.348, n = 193) as compared to MeCP2-positive neurons (0.994 ± 0.311, n = 110) (Fig. 5A).

In the hippocampal CA1 subfield, 587 neurons were re-identified successfully, with 45 % of them being MeCP2 positive. In contrast to somatosensory cortex, however, any differing redox-baseline conditions were not evident among MeCP2 deficient (0.987 ± 0.301, n = 320) and MeCP2-positive CA1 neurons (1.014 ± 0.259, n = 267) (Fig. 5B). Thereby, pointing out further brain-regional differences.

As part of these MeCP2-correlated redox analyses we also studied triple-transgenic mice which, based on cross-breeding, expressed a mitochondria-targeted catalase (mCAT). Introducing the redox-stabilizing mCAT enzyme to the mitochondrial matrix, efficiently abolished the earlier seen differences among the identified 151 MeCP2-deficient and 59 MeCP2-positive cortical neurons (Fig. 5C). In hippocampus, the analyzed 208 MeCP2-deficient and 111 MeCP2-positive CA1 neurons still did not differ in their redox-baselines when mCAT was expressed (Fig. 5D).

## 4 Discussion

Deciphering the spatiotemporal pattern of cerebral redox alterations is essential for understanding their role in RTT pathogenesis. Therefore, we performed for the first time a detailed mapping of neuronal redox conditions in *Mecp2_roGFPc* mice on the cellular level. Based on the redox-sensor expression in this unique mouse model, we rated the redox conditions in the cytosol of excitatory projection neurons. In these analyses, we succeeded to combine the advantages of 2-photon microscopy and excitation ratiometric imaging, thereby being able to conduct true quantitative analyzes of cellular redox conditions. In particular, we were able to quantify redox conditions in individual neurons embedded in their physiological tissue environment and being located beneath the superficial tissue layers suffering from tissue slicing. Various cortical and hippocampal areas were studied in early (more rostral) and late (more caudal) hippocampal slices in order to cover as many regions as possible and to unveil local differences in redox balance. Cytosol was chosen as it represents the central cell compartment harboring all other organelles, thereby constituting a major site of redox-related modulation of cellular function. Furthermore, a distinction was made between younger (p50) and more mature (>p100) mice to document any changes over time, as RTT progresses from the largely presymptomatic to the symptomatic stage.

### 4.1 Redox-mapping reveals brain regional and maturational differences

This mapping of neuronal resting baseline redox conditions unveiled clear differences among the studied brain regions even in WT mice. CA1 neurons showed the lowest degree of oxidation, whereas highest oxidation levels were usually detected in CA3 and DG. The relative oxidation levels of the cortical regions ranged in between CA1 and CA3 and differed less intensely among each other as compared to the hippocampal subfields. With cellular metabolism constituting a major source of reactive oxidants (de Almeida et al., 2022; Dröge, 2002), these brain regional differences can be considered a consequence of the specific metabolic demand of the particular brain regions as well as their specific antioxidant capacities (Aon-Bertolino et al., 2011). Furthermore, they may be a determinant of the regional sensitivity to redox modulation and the vulnerability to oxidative stress. It should be noted that we determined resting baseline conditions only. Evaluating the regional responses to redox challenge, for example by transmitter or oxidant stimulation [see (Festerling et al., 2020; Groβer et al., 2012)], may be another point of interest, which was, however, beyond the scope of the current study.

In addition to the brain regional redox differences, we detected also an age-related component, i.e. a shift of neuronal redox balance to slightly more reducing conditions with maturation. This became only detectable by the advanced roGFP imaging approach, which allows a true quantification of cellular redox conditions and hence reliable comparisons among subjects with different genotypes or age levels. The brain is particularly sensitive to oxidative challenge, which in part is due to its high degree of O_2_ utilization (Halliwell, 2006). Accordingly, this reducing shift may present a protective measure in the maturing and hence increasingly more active brain tissue. An earlier study comparing rats at ages of 5 weeks and 9 month revealed notable, complex changes in the cerebral antioxidant capacity. In detail, the activities of glutathione-S-transferase, catalase, γ-glutamylcysteine synthetase, glutathione peroxidase and superoxide dismutase were clearly increased in the more mature rat brain, as were the levels of glutathione (Kim et al., 2003). Given that similar cell-endogenous adaptations occur also in mice, they may very well explain the observed maturation-related shifts towards more reducing conditions.

### 4.2 RTT-related redox alterations

There is substantial evidence for a redox-related component of RTT. Systemic oxidative stress has been confirmed in patients (De Felice et al., 2009) as well as in brain and skeletal muscle of mouse models of RTT (De Felice et al., 2014; Gold et al., 2014). On the cellular level, an oxidative burden and redox imbalance were confirmed in cultured mouse hippocampal astrocytes (Bebensee et al., 2017) and neurons (Festerling et al., 2020; Groβer et al., 2012), patient dermal fibroblasts (Signorini et al., 2014), and very recently also in astrocytes of patient-derived cerebral organoids (Tomasello et al., 2024). For technical reasons, the majority of these earlier studies was performed on cultured and hence neonatal and/or immature preparations. Here, taking advantage of our advanced redox-indicator mouse models, we were able to extend redox imaging to the mature and symptomatic stages, while still being able to use the tissue-based preparation of acute brain slices.

The obtained reference map of the healthy, physiologically active female WT brain clearly revealed those deviations in cellular redox balance occurring under the conditions of MeCP2 deficiency. At the presymptomatic stage of p50 any signs of an oxidative burden were not evident yet. In fact, even more reducing conditions were detected in parts of the *Mecp2*^*+/−*^ hippocampus and cortex (Fig. 3, Table 1), which may be indicative of the slowed postnatal development in RTT (Einspieler and Marschik, 2019). However, with ongoing maturation and transition into the symptomatic stage, i.e. beyond p100, clearly more oxidized conditions were then observed in CA3 and DG as well as in all cortical regions of *Mecp2*^*+/−*^ mice. In line with our observations, an earlier report on a different mouse model of RTT (MeCP2-308 mice) demonstrated more oxidized conditions in the brains of one year old female *Mecp2*^*+/−*^ mice that were apparently linked to a brain area specific mitochondrial H_2_O_2_ overproduction (De Filippis et al., 2015).

An interesting finding is the fact that the more oxidized conditions in the MeCP2-deficient brain arise from the absence of the reducing redox shift that normally occurs in WT females with maturation to p100 and beyond. The detailed biochemical and physiological processes underlying these MeCP2-related deviations still need to be explored further, as well as the reasons why CA1 and subiculum were exempted from this oxidative burden. Nevertheless, neuronal function is sensitively dependent on a proper redox balance. Any shifts towards more oxidizing or more reducing conditions may therefore provoke functional disturbances, as several hundreds of (human) proteins have been proposed to be controlled by redox-alterations (Weerapana et al., 2010). Hence, even without resulting in lasting cellular damage, such redox alterations – by modulating a multitude of various cellular proteins – may substantially disturb cellular communication and responsiveness. This perfectly matches the conditions in RTT, which is not characterized by a loss of neuronal elements, but rather a state of immaturity and miscommunication of the affected neuronal networks (Armstrong, 2005; Boggio et al., 2010; Moretti et al., 2006). Accordingly, the widespread oxidizing shifts detected here in the hippocampal and cortical brain regions may well contribute to the cognitive and motor disabilities that are inherently linked to RTT.

### 4.3 Redox-mosaicism in female Mecp2^+/−^ mice

In females, each somatic cell carries two X chromosomes and the random inactivation of either the maternal of the paternal X-chromosome gives rise to a mosaicism of cells. Accordingly, under the conditions of RTT either the mutated or the intact *Mecp2* allele is transcribed. In rodents, this X-activation occurs at the embryonic late blastocyst stage (Lyon, 1961). With the late blastocyst typically containing only 100–200 cells, all cells deriving from one of these cells present the identical X-inactivation pattern. Accordingly, a highly complex, in part patchy pattern of MeCP2 expression is to be expected. In consequence, the severity of RTT manifests at varying degrees. As presented here, this also applies to cellular redox balance.

By correlative redox microscopy, we were able for the first time to correlate neuronal redox conditions in heterozygous *Mecp2*^*+/−*^ mice with cellular MeCP2 expression. The MeCP2 expression pattern in *Mecp2*^*+/−*^ mice showed marked degrees of variability among brain regions as well as among individual mice, as pointed out by others earlier (Wither et al., 2013). In the hippocampal CA1 subfield, redox conditions of MeCP2-deficient and MeCP2-positive pyramidal neurons did not differ at p50 (Fig. 5B). This very well matches the observation that in *Mecp2*^*+/−*^ mice CA1 – together with subiculum – is one of the very few brain regions not deviating to a more oxidized redox balance beyond p100. Furthermore, CA1 was the only brain region of *Mecp2*^*+/−*^ mice undergoing the maturation-related reducing shift generally observed in WT mice (Fig. 3C). It therefore seems that – against expectation – redox homeostasis is still largely intact beyond p100 in these otherwise highly vulnerable CA1 pyramidal neurons (Schmidt-Kastner and Freund, 1991; Wilde et al., 1997).

In contrast, MeCP2-deficient somatosensory cortical neurons presented a more oxidized cytosol than adjacent MeCP2-positive cells already at p50 (Fig. 5), i.e., at a largely presymptomatic stage of *Mecp2*^*+/−*^ mice (Guy et al., 2001), thereby suggesting a cell-autonomous redox imbalance in the absence of MeCP2. This confirms earlier concepts that the redox-alterations in RTT are early changes which precede the onset of typical symptoms and which may contribute to early neuronal vulnerability in a region- and cell-specific manner (De Felice et al., 2014; Groβer et al., 2012; Müller and Can, 2014), possibly even acting in synergy with a chronic subclinical inflammatory state to give rise to a condition of oxinflammation (Pecorelli et al., 2016).

Here, we recorded redox conditions in neurons only. Accordingly, our findings do not rule out that ROS are also generated by neighboring non-neuronal cells and may secondarily affect neurons as well. Along this line, we observed earlier in cultured mouse hippocampal astrocytes more oxidized cytosolic redox conditions in male *Mecp2*^*–/y*^ mice than in WT siblings (Bebensee et al., 2017), and in a patient-derived disease model of cerebral organoids, in particular astrocytic mitochondria were recently reported to show increased ROS levels which may then contribute to affect neuronal wellbeing (Tomasello et al., 2024).

Of note, expression of a mitochondrial-matrix based catalase (Schriner et al., 2005) abolished the more oxidized redox conditions seen in MeCP2-deficient somatosensory neurons. This confirms that mitochondrial targeting and a stabilization of mitochondrial function may be beneficial in RTT, as was previously demonstrated by others with a bacterial protein (CNF-1)-based strategy (De Filippis et al., 2015).

## 5 Concluding remarks and outlook

Cerebral redox-mapping in *Mecp2_roGFPc* mice revealed clear brain-regional differences in neuronal redox-baseline conditions. Furthermore, a maturational reducing shift was uncovered. Being mostly absent in *Mecp2*^*+/−*^ mice, it appears to be the primary reason for the more oxidized redox conditions manifesting in the MeCP2 deficient brain beyond p100. Correlational redox microscopy in *Mecp2*^*+/−*^ mosaic tissue detected first cell-type-specific, genotype-related oxidative changes already on p50. It therefore, would be desirable to define a time-point at which the organism is no longer able to compensate for the disturbed redox homeostasis and at which cellular redox conditions then deviate towards a pathophysiological range. Accordingly, additional time points should be addressed in future studies, and also further cellular compartments such as the mitochondrial matrix should be included. This is where our roGFPm mice (Wagener et al., 2016), which express the roGFP1 redox indicator in the mitochondrial matrix, may be of interest, especially when crossbred with *Mecp2*^*+/−*^ mice (Müller, 2019). Finally, as mCAT expression proved efficient to counteract the oxidizing shift in MeCP2-deficient neurons of somatosensory cortex, also this mitochondria-targeting approach may deserve further detailed therapeutic assessment.

## CRediT authorship contribution statement

**Hendrik Woeste:** Writing – original draft, Visualization, Investigation, Formal analysis. **Laura van Agen:** Writing – original draft, Visualization, Investigation, Formal analysis. **Michael Müller:** Writing – review & editing, Writing – original draft, Visualization, Supervision, Funding acquisition, Formal analysis, Conceptualization.

## Declaration of Generative AI and AI-assisted technologies in the writing process

Generative AI or AI-assisted technologies have not been used in the writing process of the manuscript.

## Declaration of competing interest

The authors declare that they have no known competing financial interests or personal relationships that could have appeared to influence the work reported in this paper.

## Data Availability

Data will be made available on request.
